# Seroprevalence of Hepatitis C Virus and Factors Associated with It in Armenia, 2021

**DOI:** 10.3390/v16091446

**Published:** 2024-09-11

**Authors:** Anahit Demirchyan, Antons Mozalevskis, Serine Sahakyan, Lusine Musheghyan, Lusine Aslanyan, Diana Muradyan, Narina Sargsyants, Gayane Ghukasyan, Varduhi Petrosyan

**Affiliations:** 1 Turpanjian College of Health Sciences, American University of Armenia, 40 Marshal Baghramyan Ave., Yerevan 0019, Armenia; serine_sahakyan15@alumni.aua.am (S.S.); lmusheghyan@aua.am (L.M.); laslanyan@aua.am (L.A.); dmuradyan@aua.am (D.M.); vpetrosi@aua.am (V.P.); 2 Global HIV, Hepatitis and Sexually Transmitted Infections Programmes, World Health Organization, 1211 Geneva, Switzerland; mozalevskisa@who.in; 3 National Institute of Health, Ministry of Health, Republic of Armenia, Yerevan 0051, Armenia; sknarina70@mail.ru; 4 World Health Organization Country Office in Armenia, Yerevan 0015, Armenia; ghukasyang@who.int

**Keywords:** hepatitis C virus, seroprevalence, risk factors, Armenia, general population

## Abstract

Hepatitis C virus (HCV) infection is among the leading causes of cirrhosis and hepatocellular carcinoma. Knowledge of its prevalence and risk factors can help to effectively fight the virus. This study was the first to investigate the seroprevalence of HCV, its genotypes, and factors associated with it among the general adult population of Armenia selected countrywide via cluster sampling. Anti-HCV antibodies were detected using third-generation immunoassay. Polymerase chain reaction and genotyping was performed among anti-HCV-positive individuals. Shortly after testing, the participants underwent a telephone survey. Logistic regression models were fitted to identify factors associated with anti-HCV antibody positivity and chronic HCV infection. The prevalence of anti-HCV antibodies among 3831 tested individuals was 2% (99% CI 1.4, 2.5), and chronic HCV infection was 0.7% (99% CI 0.4, 1.0), with genotypes 3 and 2 being the most common. The risk factors for chronic HCV infection included self-reported chronic liver disease (95% CI 1.47, 15.28), having tattoos (95% CI 1.34, 10.94), ever smoking (95% CI 1.16, 9.18), and testing positive for hepatitis B virus core antibody (95% CI 1.02, 7.17). These risk factors demonstrate that there could be room for strengthening infection control measures to prevent the transmission of HCV in Armenia.

## 1. Introduction

Hepatitis C virus (HCV) infection is a global concern due to its high prevalence in many regions of the world, making it one of the main causes of chronic liver disease with frequent progression to cirrhosis and hepatocellular carcinoma (HCC) [1]. It has been commonly considered that if untreated, HCV is a self-limiting disease for only 15–25% of infected people. The remaining 75–85% of infected individuals do not clear the virus and become chronic carriers [2]. However, some recent studies report considerably higher percentages of spontaneous clearance of the virus [3,4]. Approximately 20% of those chronically carrying the virus develop end-stage hepatic and extrahepatic complications over a period of 20–30 years [5,6,7]. In 2019, an estimated 290,000 people died globally from HCV infection-related causes [8]. According to the WHO Global Hepatitis Report 2017, roughly one-third of all HCV infection-related deaths are attributable to HCC and two-thirds to cirrhosis and hepatic decompensation [9]. There are several factors that contribute to the progression of chronic HCV infection to an end-stage liver disease. These include older age at the time of infection acquisition, male sex, coinfection with HIV and/or hepatitis B virus, immunosuppression, obesity, diabetes, and harmful alcohol use [5,7].

According to a recent global review and modelling study, in 2020, 56.8 million people were chronic carriers of the hepatitis C virus, which corresponds to a 0.7% global prevalence of chronic HCV infection [10]. This estimate reflects a clear decreasing trend, as the prior review and modeling study conducted by the same team in 2015 estimated the global prevalence of HCV to be 1.0%, equivalent to 71.1 million chronic carriers of the virus [11]. The decreasing trend started in the early 2000s; however, it has not been uniform across the world, being evident mainly in high-income countries with better access to curative direct-acting antiviral (DAA) therapy, whereas in some lower income countries, an opposite trend has been observed [2,12]. Nevertheless, it is important to note that at the country level and—as a result—regional and global levels, the HCV burden estimates are subject to limitation due to a lack of robust prevalence data, as only a small proportion of countries globally have ever conducted nationally representative seroprevalence studies. This is despite the fact that regular biomarker surveys have been recommended by WHO as the method of reference to estimate the prevalence of chronic infections in general populations [13].

HCV is mainly transmitted via direct exposure to blood, which occurs during unsafe injections, blood and blood components transfusion, and other blood-related procedures, commonly happening due to sharing needles and injecting equipment during drug use, tattooing, and medical procedures in settings with poor infection control [2,5,14]. Other less common transmission routes include vertical transmission from mother to infant and transmission during sexual intercourse, especially among men who have sex with men (MSM), and particularly among those living with HIV [5]. Hence, there are several population groups at higher risk of exposure to HCV and chronic HCV infection. These are people who inject drugs, MSM, people in prisons and other closed settings, and sex workers [2].

A survey of civil society organizations working in the field of hepatitis C conducted in 11 central Asian and eastern European countries during 2015–2016 found major gaps in epidemiological data on HCV in many of these countries, including Armenia. A wide spectrum of anti-HCV prevalence among the general population was reported in these countries ranging from 1.5% to 7.5% [15]. The prevalence of chronic hepatitis C in Armenia has never been measured through a representative countrywide seroprevalence study. The prevalence data based on secondary testing of frozen blood samples or screening of different population groups in Armenia have varied, from 0.5% among pregnant women to 64.0% among people who inject drugs [16]. According to estimated data, Armenia was considered a country with intermediate seroprevalence of HCV antibodies. Based on a review of various secondary prevalence data sources, and consensus expert opinion, it was estimated that the prevalence of anti-HCV positivity among the general adult population of Armenia was 4.0% in 2018 [17].

There is a viral hepatitis notification system in Armenia, according to which all identified acute and chronic HCV infections are reported to the National Center for Disease Control and Prevention (NCDC). These data indicate a clear declining trend in the incidence of acute HCV infection during the last decade in Armenia [18]. According to NCDC data, 967 cases of anti-HCV seropositivity were registered in Armenia in 2021, which is equal to an incidence rate of 32.6 per 100,000 population [18]. However, this rate is based on either symptomatic cases or routine checking for anti-HCV positivity among different patient groups and, therefore, omits the majority of incident HCV infections that are asymptomatic [19]. It is estimated that, globally, only 20% of infected individuals are aware of their infection. Moreover, only 15% of those aware of their infection actually receive treatment for it [2]. Presumably, the situation is not much different in Armenia in this regard. The rates of HCV-related hepatic and extrahepatic complications in Armenia are also unknown, making it difficult to estimate the burden of HCV. However, these data were crucially needed as Armenia joined the global strategy adopted by WHO aimed at eliminating viral hepatitis as a public health issue by 2030 [20]. This could be achieved only with careful planning and applying effective countrywide prevention, screening, and treatment interventions simultaneously [21]. For this reason, reliable data on the prevalence of HCV infection and factors associated with it were desperately needed. Therefore, this study sought to estimate the prevalence of past or present exposure to HCV, the prevalence of chronic HCV infection, and the risk factors of both outcomes among the adult population of Armenia.

## 2. Materials and Methods

### 2.1. Study Design and Sampling

A cross-sectional population-based countrywide study among the adult population of Armenia was conducted during May–September 2021. The study had two major components: laboratory testing of blood serum samples of participants for biomarkers of hepatitis C, hepatitis B, and SARS-CoV-2 viruses, and a telephone survey among them using a structured questionnaire. The study protocol was developed based on the WHO proposed template for this type of study [22]. The study included adults aged 18 years and older residing in Armenia. Probability proportional to size cluster sampling was applied to draw the study sample. All 11 administrative units of Armenia including ten provinces (marzes) and the capital city of Yerevan were covered. Primary healthcare facilities located in urban areas (polyclinics) served as the clusters for this study, considering that these facilities served both urban and rural populations. In each province and Yerevan, one-third of the polyclinics were selected from the list of all polyclinics functioning in the given area using systematic random sampling proportionate to the served population size. The study participants were then selected from the populations served by each polyclinic using simple random sampling within the national e-health operator “Armed”.

### 2.2. Sample Size and Data Collection

The study sample size was calculated using the formula for estimating a population proportion:

n = N × X/(X + N − 1), where X = Z_α/2_^2^ × p × (1 − p)/(MOE)^2^,

where N is the population size, p is the sample proportion, Z_α/2_ is the critical value of the normal distribution, and MOE is the margin of error. With the following assumptions—30% estimated prevalence of anti-SARS-CoV-2 antibodies, 1.45% margin of error, 0.05 type 1 error, and 95% confidence level—the calculated sample size was 3832. If assuming the expected prevalence of anti-HCV antibodies to be 2%, this sample size allowed calculation of the anti-HCV antibody prevalence estimate with an MOE of 0.44%. The calculated sample size was distributed among the provinces proportionate to their population size, and then equally divided between selected polyclinics in each province.

The national e-health system “Armed” (The “Armed” system is a unified health information management system in Armenia where 2.9 million citizens’ personal medical data are stored. All facilities providing state-guaranteed PHC services are attached to the “Armed” system and must use it. The personal page of the citizens/patients on the “Armed” platform contains information on the PHC facility the patient is registered at.) generated the contact information of the needed number of randomly selected participants from each polyclinic. This information was transferred to the personnel of the polyclinic, who had a legal right to contact the selected study participants and were trained to recruit participants. They contacted the potential participant by phone, introduced the study, and asked for his/her consent to participate. If agreeing to participate, the individual was invited to the clinic for blood testing. The participant recruitment for each polyclinic continued until the needed sample size was reached.

All the research sites (polyclinics) followed the same standard protocol for data collection. Blood sampling was performed in the polyclinic by a trained nurse in a well-ventilated room especially allocated for the purpose. Prior to blood sampling, participants were informed about the study details and their rights and signed a written consent form to participate. The telephone interviews took place within the first two weeks after the blood sampling. Trained interviewers conducted the interviews using a structured questionnaire. Data were collected using tablets where the Alchemer online survey tool was installed.

Each positive test result was communicated to the participant by a trained research team member (medical doctor) via a phone call and the individual was referred to the respective state-funded medical service for further counseling and treatment. Negative results were sent to the participants via SMS messages.

### 2.3. Serum Tests and Definitions

From each participant, 5–10 mL of venous blood was collected via venipuncture and immediately centrifuged for serum extraction. The serums were kept at 2–8 °C when being stored for less than seven days or at −20 °C whenever longer periods were expected before the testing could be conducted. All the samples were transported to the laboratory of the National Center for Infectious Diseases where they were tested, as this laboratory had the needed capacity and quality to perform third-generation serological and molecular testing for all the infections targeted by the study. The presence of anti-HCV antibodies was viewed as serological evidence of past or present HCV infection, while the presence of HCV RNA was viewed as evidence of chronic HCV infection [23]. The following procedure was followed: (1) all samples were tested for anti-HCV antibodies using third-generation enzyme immunoassay (Elecsys Anti-HCV-Roshe Diagnostics); (2) if anti-HCV antibodies were detected, HCV RNA tests were performed for the presence of HCV RNA and viral load (using AmpliSens HCV Monitor FL-interlabservice, Central Research Institute for Epidemiology, Moscow, Russia); and (3) if HCV RNA was positive, HCV genotyping was performed (using AmpliSens HCV Genotype 1/2/3, Central Research Institute for Epidemiology, Moscow, Russia).

### 2.4. Survey Instrument and Study Variables

Topics covered by the survey instrument included the respondent’s sociodemographic characteristics, self-reported chronic health conditions, any symptoms experienced during the last six months, knowledge and behavior on prevention of infectious diseases, as well as a few items measuring the most common risk factors of exposure to the hepatitis C virus. Respondents were also asked whether they were ever told by a doctor that they were infected with hepatitis C.

Variables included in this study were generated from both the seroprevalence study and the survey. The seroprevalence study provided the two outcome variables of this study: anti-HCV antibody status and HCV RNA status based on PCR test. Both these variables were dichotomous. The variables generated from the survey data were grouped into sociodemographic, health status, health literacy, and health risk behavior domains. The sociodemographic domain included age, sex, education, employment, residence, family size, and socioeconomic status (SES) score. The health status domain included anti-hepatitis B core antibody status, the presence of hepatitis B surface antigen (HBsAg), self-reported chronic conditions (diabetes, chronic liver disease, heart disease, obesity, cancer, chronic hematological disorder, and asthma), and symptoms experienced during the last six months (vomiting, fatigue, loss of appetite, nausea, abdominal pain, fever, and diarrhea). The health literacy domain included knowledge on the prevention of infectious diseases and difficulty in understanding health-related information. The health risk behavior domain included smoking (ever, current), having tattoos, undergoing a blood transfusion, ever having been imprisoned, and frequency of visiting a dentist.

### 2.5. Analysis

We used IBM SPSS Statistics for Windows, Version 21 for the analysis. Proportions and 99% confidence intervals were calculated for positivity to anti-HCV antibodies and HCV RNA status to estimate the prevalence of past/present exposure to HCV infection and the prevalence of chronic HCV infection, respectively. To generate more precise prevalence estimates for the population of Armenia, the descriptive analysis was carried out using weighting by sex and 5-year age groups to compensate for differences in age and sex distribution between the study sample and the general population of Armenia, which were attributable to different response rates among different age and sex groups. Independent variables were analyzed descriptively using frequencies and proportions for categorical variables, and means and standard deviations for continuous variables. Proportions and means were compared between the groups with different HCV outcomes using Χ^2^ and *t*-tests, respectively. This was followed by univariate and then multivariable logistic regression analysis between independent variables and each of the two outcome variables. Most of the study’s variables were dichotomous. For the few categorical variables, dummies were created to be included in the regression analysis. Continuous variables were entered into the regression analysis after checking the linearity of their association with each outcome variable on the logistic scale. As the final step, logistic regression models were fitted to identify risk factors of each outcome among the adult population of Armenia. The fit of the models was tested by the Hosmer and Lemeshow goodness of fit test and area under the Receiver Operating Characteristic (ROC) curve [24,25]. The final models were checked for collinearity using Variance Inflation Factor (VIF) statistics.

## 3. Results

### 3.1. Response Rate and Study Sample

Of a total of 9009 eligible individuals contacted and invited to their respective PHC facility for blood testing, 3831 (42.5%) accepted the invitation and underwent blood testing for HCV. Of them, 3476 individuals (90.7%) participated in the phone survey. The numbers of participants from the provinces and Yerevan city were proportionate to their population sizes and constituted around 0.13% of the total population in each. Due to different response rates among different sex and age groups, males were underrepresented in the study sample and accounted for 30.0% of the total participants, while they accounted for 47.2% of the population of Armenia. Similarly, the age groups below 30 and over 75 years old were underrepresented in the study sample. Therefore, all the prevalence estimates of HCV infection were calculated using weighting by sex and 5-year age groups.

### 3.2. HCV Prevalence and Descriptive Findings

**Figure 1:**
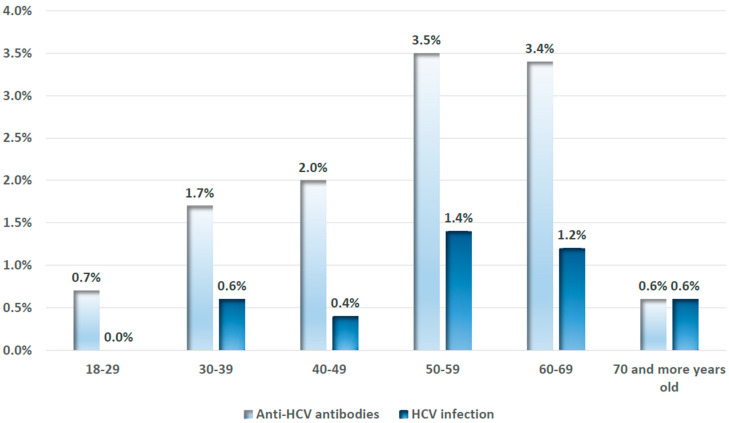
Weighted prevalence estimates of anti-HCV antibodies and chronic HCV infection by 10-year age groups among the study participants, Armenia, 2021.

The prevalence of anti-HCV antibodies, indicating past or present exposure to HCV infection, was 2.0% with the 99% confidence interval (CI) ranging from 1.4% to 2.5%. The overall prevalence of HCV RNA—the marker of chronic HCV infection—was 0.7% (99% CI 0.4%; 1.0%). Hence, the viraemia rate among those ever exposed to HCV was 35%. The prevalences of both antibodies and chronic infection were higher among 50–69 years old (3.4% and 1.3%, respectively). The weighted prevalence estimates of being exposed to HCV and having chronic HCV infection by 10-year age groups are presented in Figure 1.

The HCV genotyping identified five individuals with genotype 1, ten individuals with genotype 2, and ten individuals with genotype 3. The weighted percentage estimates for this distribution were: 41.7% for genotype 3; 37.5% for genotype 2; and 20.8% for genotype 1. It is noteworthy that only 21.9% of those testing positive for HCV antibodies and 20.0% of those carrying HCV based on PCR test results reported that they have ever been told by a doctor or other health worker that they had hepatitis C.

In the weighted analysis, the study variables were compared between the groups exposed and unexposed to HCV infection, as well as between those with and without chronic HCV infection. Table 1 and Table 2 report the results.

Those testing positive for anti-HCV antibodies were generally older than those testing negative. The same was true for those carrying the virus. The proportion of males was higher in the group exposed to the virus compared to those unexposed, and in the group positive to HCV PCR test compared to those negative to it. The proportion of employed (including students) was significantly lower in the groups exposed to HCV or carrying the virus compared to the comparison groups. The proportion of those living in Yerevan was significantly higher in both exposed and infected groups compared to those unexposed and free from infection. Of the health status variables, factors significantly different between the groups exposed and unexposed to HCV infection included being positive for the anti-hepatitis B core antibody, and reporting diabetes, chronic liver disease, and symptoms of diarrhea (all were more frequent in the exposed group). Similarly, all these factors except diabetes were significantly more common among HCV carriers compared to non-carriers (Table 2). No differences between the groups were found with regard to health literacy variables. In contrast, the groups were different in terms of a number of behavioral variables. Current smoking was more frequent among those exposed to the virus compared to those unexposed. Smoking ever in life was significantly more common among both groups exposed to the virus and having chronic HCV infection. Both these groups reported having tattoos more frequently than the comparison groups. The participants having anti-HCV antibodies reported ever undergoing blood transfusion more frequently than those unexposed to the virus (Table 1). Those carrying the virus reported being imprisoned significantly more commonly than those uninfected (10.5% versus 1.6%, *p* = 0.040). The groups of exposed and unexposed to HCV infection were different also in terms of frequency of visiting a dentist. The exposed group used dental services less frequently than the unexposed group (Table 1).

### 3.3. Factors Associated with HCV Status

The fitted logistic regression model identified the following risk factors for exposure to HCV—male sex, Yerevan residence, being unemployed/retired, undergoing blood transfusion, having tattoos, and reporting chronic liver disease (Table 3). When holding all the other variables constant, male sex was associated with a 3.2 times higher likelihood of being exposed to HCV than female sex. Compared to residing in marzes, living in Yerevan increased the odds of being exposed to HCV infection 1.8 times. Being employed or a student was associated with two times lower odds of being exposed to the virus compared to being unemployed or retired. Having a blood transfusion ever in life increased the odds of exposure to HCV infection 4.8 times and having tattoos increased its odds twice. Reporting chronic liver disease was associated with a 3.8 times higher likelihood of having been exposed to the HCV virus.

The fitted model also included two marginally significant variables—testing positive for the hepatitis B virus (HBV) core antibody and having asthma requiring medication—both positively associated with the odds of having been exposed to HCV. The model had acceptable fit indices (Table 3) and no issues with collinearity (all VIF values were below 1.1).

Several logistic regression models of determinants of chronic HCV infection with somewhat different sets of predictors were fitted and one of these models slightly exceeded the others in its fit indices; thus, it was selected as the final model and is displayed in Table 4. According to the model, four determinants of chronic HCV infection were identified: self-reported chronic liver disease, having tattoos, smoking ever in life, and testing positive for the hepatitis B core antibody. Reporting chronic liver disease was associated with 4.7 times higher odds of carrying HCV. Having tattoos increased the likelihood of chronic HCV infection 3.8 times. Regular smoking ever in life increased the odds of having chronic HCV infection 3.3 times. Being exposed to HBV ever in life, measured by the presence of anti-HBV core antibodies, was associated with 2.7 times higher likelihood of carrying HCV infection.

The model also included self-reported presence of chronic fatigue, which was marginally significantly positively related to chronic HCV infection. The model had good fit indices (Table 4). All VIF values were below 1.1 indicating no issues with collinearity.

## 4. Discussion

This study was the first attempt to estimate the prevalence of HCV markers among the general population of Armenia using a rigorous methodology and a representative population sample. All prior attempts of estimating HCV prevalence were restricted to the use of data on screening for HCV infection partially available for some high-risk groups (e.g., healthcare providers, patients with sexually transmitted infections, neoplasms, tuberculosis, hemodialysis patients, people who use drugs, and prisoners) or secondary testing of frozen blood samples of different groups of children and adults, primarily collected for other purposes [16]. According to these prior reports, the estimates of HCV prevalence among different population groups in Armenia (based on anti-HCV antibodies) ranged from 0.5% among pregnant women and 1.5% among blood donors to as high as 39.3% among prisoners and 64.0% among people who inject drugs [16]. The estimate for the general adult population varied from 2.9% [16] to 4.0% [17,26]. The current population-based seroprevalence study found a considerably lower prevalence of anti-HCV (antibodies) among the general adult population (2.0%), and even its 99% confidence interval (1.4%, 2.5%) did not include the lowest prior estimate. The prevalence of anti-HCV in Armenia found in this study is lower than the recent estimate for the prevalence of anti-HCV antibodies in Eastern Europe (3.3%) [15]. However, there is a possibility that the groups at higher risk for HCV and those with HCV complications were underrepresented in our study sample. The reasons for this could be both the difficult-to-reach character of many of these groups (people who inject drugs, imprisoned people, sex workers), as well as the health condition of some others (patients with sexually transmitted infections, neoplasms, tuberculosis, complications of chronic HCV infection, and hemodialysis patients) that might have prevented them from joining our study sample. HCV prevalence estimates from general population surveys usually underestimate the national prevalence because these surveys are seriously limited in covering the population groups at the highest risk of HCV infection [27]. A study in the US estimated that, because of the low coverage of some high-risk population groups, national surveys have undervalued the true prevalence of HCV by 36.5% [28]. In this study, the prevalence of HCV was the highest among 50–69 years old. Considering a quite similar HCV prevalence pattern in the US, in 2012, the Centers for Disease Control and Prevention recommended one-time HCV testing for all US residents born during 1945–1965 [29].

According to different studies, the proportion of HCV-exposed subjects who develop chronic infection varies widely, from 30% to 80% [3,4,30]. In this study, the proportion of infected among those exposed to HCV was 35%. The HCV genotype distribution identified in this study (almost equally prevalent genotypes 3 and 2 followed by twice less prevalent genotype 1) was also somewhat different from the genotype distribution reported in the prior research in Armenia, according to which genotypes 1 and 3 were the most prevalent followed by a considerably less prevalent genotype 2 [31]. However, given the small number of cases within each genotype group (10 or less) in the current study sample, the described difference could be largely attributable to chance.

This study identified sets of predictors of anti-HCV positivity and chronic HCV infection in Armenia, which, as expected, considerably overlapped with each other. In particular, self-reported chronic liver disease, having tattoos, and being ever exposed to HBV infection were among the predictors of both outcomes. Generally, the identified predictors were related to either behaviors or procedures that increase the risk of infection with HCV (tattoos, blood transfusion), factors related to a higher chance of practicing higher-risk behaviors (male sex, unemployment, living in Yerevan, smoking), and conditions possibly related to HCV infection (chronic liver disease, fatigue) or infections with similar transmission routes (exposure to HBV).

The role of cosmetic and other blood-related procedures in transmitting HCV is well established in the literature. Tattooing, piercing, and injection drug use are considered to be among key behavioral risk factors for acquiring HCV [32]. Our finding of tattooing being related to a twice higher likelihood of being exposed to HCV and 3.8 times higher likelihood of being HCV-infected is consistent with the finding of a study in Malaysia that identified 3.7 times higher odds of HCV infection associated with tattooing [33]. The same study found that undergoing blood product transfusion before 1992 was associated with seven times higher odds of being infected with HCV in Malaysia [33]. Consistent with this, we found an almost five times higher likelihood of HCV exposure among those who underwent blood transfusion ever in life. This study did not set a time boundary for this variable; therefore, we cannot relate this finding to an earlier period in Armenia when testing of blood products was mainly conducted with second-generation tests having insufficient sensitivity to capture low quantities of viral antibodies [34]. A recent evaluation of the viral hepatitis response in Armenia conducted in November 2022 by the WHO and Robert Koch Institute (RKI) concluded that the blood products were safe and the blood testing algorithms were up to date at the Haematology Centre named after R. Yeolyan in Yerevan, but not yet in the remaining centers of Armenia. Therefore, the mission recommended implementing systematic quality assurance mechanisms in all centers dealing with blood products and securing high-quality services across the country [18].

Some of the determinants of being exposed or infected with HCV found in this study may possibly be related to having a higher probability of practicing higher-risk behaviors. These characteristics included male sex, unemployment, smoking, and residing in the capital city of Yerevan. A meta-analysis of 150 studies on gender differences in risk-taking tendencies found that, generally, men are prone to take greater risks than women, including drug use and unprotected sexual activities [35]. This study lacked the advantage of collecting data on participants’ injection drug use and higher-risk sexual behaviors that are known determinants of HCV transmission [33,36,37]. Yet, male gender could indirectly point out the association between the study outcomes and these well-established risk factors of HCV transmission as, compared to women, these behaviors are more pronounced among men [35].

Unemployment, which was related to a higher chance of having been exposed to HCV in this study, has also been shown to be associated with overexposure to some risky behaviors, such as smoking, harmful alcohol use, etc. [38]. Importantly, unemployment was found to be related to increased sexual risk-taking in adolescent men [39]. Moreover, unemployment was associated with co-occurrence of several risky behaviors [38]. Notably, unemployment was among independent predictors of anti-HCV positivity in a recent study in Georgia as well [40]. Similarly, cigarette smoking was found to be related to multiple health risk behaviors, especially among adolescents. These behaviors included higher use of different drugs, alcohol abuse, early sexual intercourse, and early pregnancy [41,42]. This could explain the relation we found between tobacco smoking—an indirect evidence of the higher probability of practicing other risky behaviors related to HCV transmission—and being infected with HCV. The last risk factor in this group of “indirect” predictors was residing in a highly urbanized area (Yerevan city versus provinces), which was found to be associated with a higher likelihood of being exposed to HCV. Consistent with this, there are some reports on the higher prevalence of HCV infection in urban versus rural areas [43]. However, some other studies did not find any substantial difference between rural and urban populations in terms of the prevalence of anti-HCV positivity [44]. Possibly, this conflicting evidence reflects the sociocultural differences between the compared settings that could be location-specific.

The last group of factors associated with HCV status in this study included coinfection with HBV and self-reported chronic liver disease. Coinfection of HCV and HBV is not uncommon because of the same transmission routes of both viruses, although the existing estimates of the prevalence of coinfection vary widely across studies, reaching the highest values in highly endemic areas and among high-risk population groups [45]. In this study, we found a high rate (28.8%) of the co-occurrence of HCV infection and positivity to the anti-HBV core antibody. Yet, the HCV and HBV (measured by HBsAg positivity) coinfection rate was not that high (4.0%). This may be explained by the fact that in the case of HCV and HBV coinfection, HCV is usually overt, while HBV is occult, meaning that the serum does not contain HBsAg and HBV DNA, but they are detectable in the liver tissue [46]. Self-reported chronic liver disease, which was strongly related to both HCV exposure and being infected with the virus, most probably, was the direct sequence of HCV infection, which, together with HBV, accounts for the majority of cirrhosis and hepatocellular carcinoma cases throughout the world [47]. It is known that one-third of patients chronically infected with HCV develop progressive liver injury over a period of 20–30 years and, therefore, suffer from chronic liver disease [48]. The findings of this study on the possible positive association between fatigue and HCV infection, as well as asthma and HCV exposure, also deserve attention. Higher levels of fatigue among patients with chronic HCV infection were reported in a review article as one of the extrahepatic manifestations of HCV [49]. It is also known that immune-related mechanisms underlie some extrahepatic manifestations of HCV, and this could explain the possible link between asthma and HCV found in this study [50].

As mentioned above, for feasibility reasons, this study collected data only on a few possible determinants of HCV infection, omitting some important risk factors, such as injection drug use, piercing, sexual behavior, coinfection with HIV, receiving immunosuppressive therapy, age at infection, host genetic factors, and undergoing major surgical procedures [14,23,33,51,52]. However, the determinants of HCV status found in this study indirectly pointed out the possible role that many of these unmeasured factors play in the transmission and progression of HCV infection. Additionally, the finding of HCV genotype 3 being the most prevalent in this study population possibly indicates the considerable role of injection drug use in spreading the virus, as genotype 3 was found to be the predominant genotype among people who inject drugs globally [53]. Another limitation of this study might be the possible underrepresentation of some difficult-to-reach population groups at high risk for HCV infection in the study sample. Yet, this is only a presumption with no measurable evidence. The study made thorough efforts to generate a representative sample. Still, the low rate (42.5%) of those who accepted the invitation for undergoing blood testing allows for this presumption.

## 5. Conclusions

This was the first study in Armenia that provided robust estimates of the prevalence of HCV infection ever in life and chronic HCV infection among the general adult population. Its findings revealed a lower-than-expected prevalence of both chronic HCV infection (0.7%, 99% CI 0.4%; 1.0%) and past or present exposure to the virus (2.0%, 99% CI 1.4%; 2.5%). However, these estimates indicate that over 16,300 adults in Armenia (given the adult population size of 2.33 million) live with chronic HCV infection. Importantly, eighty percent of those infected with HCV were unaware of their infection status in this study. This is closer to the highest limit of the range of undiagnosed HCV infection globally, estimated to vary from 45% to 85% [54]. This fact underlines the importance of achieving better detectability of those infected with HCV to facilitate their timely DAA treatment, preventing the life-threatening complications of the infection [55,56,57]. Public education interventions on how to avoid risky behaviors for contracting the virus also have significant potential to reduce the transmission of HCV, especially among the high-risk groups in the population [58]. In order to prevent the transmission of HCV during medical, cosmetic, and other blood-related procedures, strengthening infection prevention and control measures is recommended. We hope that the estimates found in this study on the prevalence of exposure to HCV and HCV infection among the general adult population of Armenia could potentially also benefit the wider community, contributing to more robust prevalence estimates on regional and global levels.

## Figures and Tables

**Table 1:** Distribution of selected variables by participants’ anti-HCV antibody status, Armenia, 2021.

	N *	Anti-HCV Antibodies		*p*-Value	Total (n = 3474) *
		Positive (n = 69) *	Negative (n = 3405) *		
*Socioeconomic variables:*					
Age, mean (SD)	3473	50.0 (12.9)	45.7 (17.4)	0.043	45.8 (17.3)
Socioeconomic status score, mean (SD)	2696	46.2 (31.2)	49.9 (35.3)	0.452	49.9 (35.2)
Family size, mean (SD)	3451	4.4 (2.0)	4.4 (1.8)	0.912	4.4 (1.8)
Sex: Female, %	3473	27.9	55.1	<0.001	54.5
Male, %		72.1	44.9		45.5
Education: Less than university, %	3454	58.5	57.7	0.999	57.8
University/higher, %		41.5	42.3		42.2
Employment: Employed/student, %	3437	29.7	56.9	<0.001	56.4
Unemployed/seasonal migrant, %		48.4	28.3		28.7
Retired/disabled, %		21.9	14.8		15.0
Residence: Yerevan city, %	3473	47.1	36.0	0.074	36.3
Marzes (provinces), %		52.9	64.0		63.7
*Health status variables:*					
Anti-Hepatitis B core antibody positive, %	3475	24.6	13.3	0.011	13.5
Hepatitis B surface antigen (HBsAg) positive, %	3474	2.9	0.7	0.097	0.8
Diabetes, %	3395	17.2	7.6	0.015	7.8
Chronic liver disease, %	3391	17.2	2.6	<0.001	2.9
Obesity, %	3384	6.3	10.2	0.403	10.1
Heart disease, %	3389	17.2	14.7	0.593	14.8
Cancer, %	3395	3.1	1.5	0.257	1.5
Chronic hematological disorder, %	3390	4.7	1.9	0.123	1.9
Asthma requiring medication, %	3390	2.2	6.3	0.054	2.2
*Symptoms experienced during the last six months:*					
Vomiting, %	3387	6.3	4.4	0.363	4.5
Fatigue, %	3391	36.5	45.6	0.162	45.4
Loss of appetite, %	3387	15.9	13.6	0.578	13.6
Nausea, %	3385	17.5	14.1	0.464	14.2
Abdominal pain, %	3383	14.3	10.9	0.413	11.0
Fever, %	3395	33.3	24.1	0.102	24.2
Diarrhea, %	3388	22.2	13.0	0.038	13.2
*Health literacy variables:*					
Contagious disease knowledge score, mean (SD)	3398	2.2 (1.4)	2.4 (1.2)	0.206	2.4 (1.2)
Health information understanding score, mean (SD)	3005	52.4 (10.2)	54.2 (10.1)	0.185	54.1 (10.1)
*Health behavioral variables* :					
Current smoking, %	3390	40.6	21.0	0.001	21.4
Ever smoking, %	3395	59.4	34.3	<0.001	34.7
Having tattoos, %	3380	20.3	10.2	0.020	10.4
Undergoing blood transfusion, %	3378	9.7	3.3	0.018	3.4
Ever having been imprisoned, %	3387	4.8	1.6	0.088	1.7
Visiting a dentist: Once in 3–5 years/more, %	3329	57.4	71.7	0.021	71.4
Less frequently/never, %		42.6	28.3		28.6

N, number of valid responses; HCV, hepatitis C virus; SD, standard deviation. * Weighted estimates are provided; *p*-value: two-sided. The names of variable groupings are provided in underlined italics.

**Table 2:** Distribution of selected variables by participants’ chronic HCV (PCR-detected RNA) status, Armenia, 2021.

	N *	Chronic HCV		*p*-Value	Total (n = 3474) *
		Yes (n = 24) *	No (n = 3450) *		
*Socioeconomic variables:*					
Age, mean (SD)	3473	53.1 (12.6)	45.7 (17.4)	0.040	45.8 (17.3)
Socioeconomic status score, mean (SD)	2696	55.8 (50.3)	49.8 (35.2)	0.558	49.9 (35.2)
Family size, mean (SD)	3451	4.5 (2.4)	4.4 (1.8)	0.829	4.4 (1.8)
Sex: Female, %	3474	25.0	54.7	0.006	54.5
Male, %		75.0	45.3		45.5
Education: Less than university, %	3455	50.0	57.8	0.519	57.7
University/higher, %		50.0	42.2		42.3
Employment: Employed/student, %	3437	30.0	56.6	0.049	56.4
Unemployed/seasonal migrant, %		50.0	28.5		28.6
Retired/disabled, %		20.0	14.9		15.0
Residence: Yerevan city, % Marzes (provinces), %	3474	41.7 58.3	36.2 63.8	0.671	36.3 63.7
*Health status variables:*					
Anti-Hepatitis B core antibody positive, %	3474	29.2	13.4	0.034	13.5
Hepatitis B surface antigen (HbsAg) positive, %	3474	4.2	0.8	0.171	0.8
Diabetes, %	3395	10.0	7.8	0.666	7.8
Chronic liver disease, %	3391	19.4	2.8	0.003	2.9
Obesity, %	3384	9.5	10.1	0.999	10.1
Heart disease, %	3390	28.6	14.7	0.112	14.8
Cancer, %	3395	5.0	1.5	0.266	1.5
Chronic hematological disorder, %	3390	4.8	1.9	0.335	1.9
Asthma requiring medication, %	3392	9.5	2.2	0.081	2.3
*Symptoms experienced during the last six months:*					
Vomiting, %	3387	0.0	4.5	0.999	4.5
Fatigue, %	3391	68.4	45.3	0.062	45.4
Loss of appetite, %	3388	15.8	13.7	0.737	13.7
Nausea, %	3384	15.8	14.2	0.744	14.2
Abdominal pain, %	3382	26.3	10.9	0.050	11.0
Fever, %	3394	36.1	24.2	0.428	24.2
Diarrhea, %	3388	31.6	13.1	0.030	13.2
*Health literacy variables:*					
Contagious disease knowledge score, mean (SD)	3398	1.9 (1.4)	2.4 (1.2)	0.082	2.4 (1.2)
Health information understanding score, mean (SD)	3005	52.4 (11.5)	54.2 (10.1)	0.479	54.1 (10.1)
*Health behavioral variables* :					
Current smoking, %	3390	35.0	21.3	0.167	21.4
Ever smoking, %	3395	65.0	34.5	0.008	34.7
Having tattoos, %	3380	35.0	10.2	0.003	10.4
Undergoing blood transfusion, %	3380	4.8	3.5	0.524	3.5
Ever having been imprisoned, %	3368	10.5	1.6	0.040	1.7
Visiting a dentist: Once in 3–5 years/more, %	3330	57.1	71.5	0.151	71.4
Less frequently/never, %		42.9	28.5		28.6

N, number of valid responses; HCV, hepatitis C virus; SD, standard deviation. * Weighted estimates are provided; *p*-value: two-sided. The names of variable groupings are provided in underlined italics.

**Table 3:** Logistic regression model of determinants of HCV exposure (anti-HCV positivity) in Armenia, 2021 (valid N = 3328).

Characteristics	OR	95% CI	*p*-Value
Male sex	3.19	1.84–5.52	<0.001
Blood transfusion ever	4.79	2.26–10.16	<0.001
Chronic liver disease	3.40	1.77–8.14	0.001
Employed vs. unemployed/retired	0.46	0.26–0.79	0.005
Living in Yerevan vs. marzes	1.82	1.09–3.06	0.022
Having tattoos	2.06	1.06–4.02	0.033
Positive for HBV core antibody	1.79	0.99–3.22	0.052
Having asthma requiring medication	2.64	0.95–7.38	0.063
Model fit statistics:	Hosmer and Lemeshow goodness of fit test, *p* = 0.187		
	Area under the ROC curve = 0.772		
	Pseudo R^2^ = 0.122		

N, number of cases; OR, odds ratio; CI, confidence interval; HBV, hepatitis B virus.

**Table 4:** Logistic regression model of determinants of chronic HCV infection (PCR-confirmed) in Armenia, 2021 (valid N = 3349).

Characteristics	OR	95% CI	*p*-Value
Chronic liver disease	4.74	1.47–15.28	0.009
Having tattoos	3.83	1.34–10.94	0.012
Smoking ever	3.26	1.16–9.18	0.025
Positive for HBV core antibody	2.71	1.02–7.18	0.045
Having fatigue	2.25	0.86–5.88	0.097
Model fit statistics:	Hosmer and Lemeshow goodness of fit test, *p* = 0.938		
	Area under the ROC curve = 0.787		
	Pseudo R^2^ = 0.134		

N, number of cases; OR, odds ratio; CI, confidence interval; HBV, hepatitis B virus.

## Data Availability

The dataset presented in this article is not readily available because the data are part of an ongoing study. Requests to access the datasets should be directed to Anahit Demirchyan (ademirch@aua.am).
